# Acetylation increases expression, interaction with TRAPPC4 and surface localization of PD-L1

**DOI:** 10.1007/s12672-023-00766-4

**Published:** 2023-08-21

**Authors:** Maria Anele Romeo, Maria Saveria Gilardini Montani, Roberta Santarelli, Rossella Benedetti, Andrea Arena, Mara Cirone

**Affiliations:** Department of Experimental Medicine, “Sapienza” University of Rome, Viale Regina Elena 324, 00161 Rome, Italy

**Keywords:** PD-L1, Acetylation, Pancreatic cancer, HDACi, VPA, JQ-1, TRAPPC4, Cytokines

## Abstract

PD-L1 is an immune checkpoint inhibitor, whose surface expression may be exploited by cancer cells to escape T cell-mediated immune recognition. PD-L1 expression and nuclear localization can be affected by epigenetic modifications, such as acetylation. In this study, we showed that VPA, a class I/IIa HDAC inhibitor, upregulated PD-L1 expression on the surface of pancreatic cancer cells. To this effect contributed the increased transcription, in correlation with histone acetylation of the PD-L1 gene and the acetylation of PD-L1 protein, which led to an increased interaction with TRAPPC4, molecule involved in PD-L1 recycling to the cell membrane. Interestingly, the BRD4 inhibitor JQ-1, counteracted PD-L1 transcription and reduced its surface expression, suggesting that such a combination could improve the outcome of VPA treatment, also because it increased the cytotoxic effect of VPA. Also considering that this HDACi did not upregulate PD-L2 and that the supernatant of VPA-treated cancer cells did not increase PD-L1 expression on the surface of macrophages exposed to it.

**Supplementary Information:**

The online version contains supplementary material available at 10.1007/s12672-023-00766-4.

## Introduction

Programmed death ligand-1 (PD-L1), encoded by the CD274 gene, is an immune checkpoint inhibitor that, when expressed on the surface of tumor or on antigen presenting cells (APC), binds to programmed death-1 (PD-1) on T lymphocytes, inhibiting their immune function. Such interactions activate Src homology region 2 domain-containing phosphatases (SPH2), leading to suppression of the T-cell receptor (TCR) signaling pathway [1]. Blocking the PD-L1/PD-1 interaction represents a promising strategy to restore T cell immunity and improve the outcome of several anti-cancer therapies [2]. One of the biomarkers predicting the success of anti-PD-L1/PD-1 treatment is high expression of PD-L1 on the cancer cell surface. However, also taking this aspect into consideration, the improvement of anti-cancer therapies so far achieved by blocking PD-L1/PD-1 interaction in cancer patients remains partial, suggesting that more in-depth investigations on the regulation of these molecules is needed to optimize the treatment [3]. PD-L1 has been reported to bind to the trafficking protein particle complex subunit (TRAPPC) 4, which plays an important role in PD-L1 endosomal recycling to the cell membrane and in preventing its lysosomal degradation [4]. Interestingly, while the increase in PD-L1 expression on the cell surface may be exploited to improve the outcome of anti-cancer treatments, its nuclear localization may reduce the efficacy of the PD-L1/PD-1 blockade. PD-L1 may act as a transcription factor and upregulate molecules that further induce immune suppression, such as programmed death ligand-2 (PD-L2) [5]. Therefore, it is important to explore not only the expression level of PD-L1 but also its subcellular localization and understand how it is regulated, particularly in the course of anti-cancer treatments.

Post-translational modifications such as acetylation may influence the stability and cellular localization of several proteins involved in oncogenesis [6]. Regarding PD-L1, a recent study has shown that it can be acetylated at Lys 263 by p300 and deacetylated by HDAC2, and that such epigenetic modification interferes with PD-L1 nuclear import and the transcription of genes that it regulates [7].

HDACi are a novel class of drugs whose anti-cancer activity has been largely demonstrated, also against pancreatic cancer, which is characterized by high aggressiveness and poor response to therapies [8, 9]. By inhibiting the activity of HDACs, these treatments enhance acetylation of histones and non-histone proteins, modulating DNA transcription and the expression and function of a variety of proteins [10]. Notably, a key role in the epigenetic regulation of protein expression is also played by the readers of acetyl lysine sites, namely the bromodomains (BRDs), particularly BRD4, which binds acetylated lysine on histones and other nuclear proteins and promotes gene transcription by RNA polymerase II (Pol II) [11]. Based on this background, in this study we investigated whether Valproic acid (VPA), a class I/IIa HDAC inhibitor that has been shown to inhibit HDAC2 [12], could influence the expression and the acetylation of PD-L1 and whether acetylation could affect its interaction with molecules such as TRAPPC4 and its localization on cell surface of pancreatic cancer cells. We chose VPA because we have previously reported that it efficiently impaired pancreatic cancer cell survival and potentiated the cytotoxic effect of the PARP-1 inhibitor AZD2461 [9, 13, 14]. Next, we investigated whether JQ-1, a small molecule that targets BRD4 and modulates the transcriptional machinery at acetylated loci [15], could counteract the effects induced by VPA.

As the release of several cytokines by cancer cells has been reported to negatively influence the efficacy of PD-L1/PD-1 blockade therapy [16], we also investigated the pattern of cytokines released by VPA-treated pancreatic cancer cells. Finally, given that macrophages represent the most numerous immune cells infiltrating the tumor bed and their activity may strongly influence the course of cancer [17], also depending on the expression of PD-L1 [18], we next evaluated whether the supernatant VPA-treated pancreatic cancer cells could affect the expression of this checkpoint inhibitor on the surface of these cells.

## Material and methods

### Cells maintenance and treatments

PaCa44 and PT45 cell lines (pancreatic ductal adenocarcinoma) (kindly provided by Dr. M. von Bulow University of Mainz and Dr. H. Kalthoff University of Kiel, Germany) were maintained as already described [19]. Cells, plated at a density of 2 × 10^5^ cells/well in 2 mL of complete medium the day before, were treated with valproic acid (VPA) (10 mM) (Sigma Aldrich, Burlington, MA, USA) for 24 and 48 h (h). Supernatants were collected and stored at − 80 °C. In some experiments, the cells were treated with VPA (10 mM) and JQ-1 (500 nM) (MedChemExpress, NJ, USA) or co-treated with VPA and JQ-1 for 48 h. To evaluate the effects of other HDAC inhibitors on PD-L1 cell surface expression, the pancreatic cell lines were treated with sodium phenylbutyrate (PBA) (Pan-HDAC inhibitor) (10 mM) (Sigma-Aldrich, Burlington, MA, USA) or nicotinamide (NAM) (Pan-Sirtuin inhibitor) (15 mM) (Sigma-Aldrich, Burlington, MA, US) for 48 h. In all the experiments untreated cells were used as controls (CT).

### Monocyte isolation, macrophage differentiation and culture with supernatant of VPA-treated and untreated PaCa44 cells

Human peripheral blood mononuclear cells (PBMCs) were isolated from buffy coats of healthy donors and monocytes were isolated by immunomagnetic cell separation as previously described [20].

To induce macrophage differentiation, purified monocytes were cultured at a density of 2 × 10^6^ cells/ml in 6-well plates in complete medium with the addition of recombinant human macrophage-colony stimulating factor (M-CSF) at a concentration of 50 ng/ml (Miltenyi Biotec, 130-093) for 6 days. Then, macrophages were cultured for 24 h in complete medium with 25% vol/vol of supernatant of VPA-treated or untreated PaCa44 cells to evaluate PD-L1 membrane expression on macrophages using FACS.

### Trypan blue assay

To evaluate the survival rate after treatment or co-treatment with VPA and JQ-1 for 48 h, a Trypan blue exclusion assay (Sigma Aldrich, Burlington, MA, USA) was used as previously described [9].

### RNA isolation and quantitative real time polymerase chain reaction (qRT-PCR)

Total RNA extraction, cDNA Reverse Transcription and qRT-PCR for PD-L1 and TRAPPC4 genes were performed as already reported [21].

### FACS analysis

Treated and untreated cells were stained with PE Human IgG1 isotype control, PE anti-human CD274 (403504 and 393608, BioLegend, San Diego, California, USA), or PE anti-human CD273 (345505, BioLegend, San Diego, California, USA) and analyzed using FACSCalibur using CELLQuest software (BD Biosciences, San Jose, CA, USA). At least 10 × 10^3^ events were recorded for each sample.

### PD-L1 K263R transfection

PaCa44 cells were seeded in 6-well plates as above reported or in a medium flask at a density of 1 × 10^6^ cells/flask. The day after, cells were transfected with empty vector or PD-L1 K263R plasmid (kindly regarded by Dr. Yang Gao) using Lipofectamine 3000 (Invitrogen, Waltham, MA, USA), according to the manufacturer’s instructions. After 24 h of transfection, cells were treated for an additional 48 h with VPA, JQ-1, or both drugs. The cells were then recovered to perform subsequent analyses.

### TRAPPC4 silencing

PaCa44 cells were seeded in 6-well plates and, the following day, transfected with TRAPPC4 siRNA (sc-96374; Santa Cruz Biotechnology) using INTERFERin^®^ (Polyplus-transfection), as described [21]. After 24 h of transfection, the cells were treated with VPA (10 mM) for 48 h and then used for other analyses.

### Western blot analysis

Following transfection and treatments, cells were lysed and 12 μg of proteins were subjected to electrophoresis and transferred on nitrocellulose membranes as previously described [22]. Membranes were then probed with specific antibodies, and developed using ECL Blotting Substrate (Advansta).

### Nuclear/cytoplasmic fractionation

To perform nuclear/cytoplasmic fractionation, 2 × 10^6^ PaCa44 cells were treated with VPA (10 mM) for 48 h. Collected cells were then centrifuged at 600*g* for 5 min at 4 °C and then lysed, first with CEB-A mix and CEB-B mix for the recovery of cytoplasmic fraction, and finally with NEB-mix for the recovery of nuclear cytoplasmic according to the manufacturer’s instructions (K266-25; Biovision Waltham, MA, USA). Proteins derived from the two different fractions were subsequently analyzed by western blot analysis.

### Immunoprecipitation assay

To perform the immunoprecipitation assay, 1 × 10^7^ PaCa44 cells were treated and/or transfected, as described above. After treatment, cells were collected, lysed in 500 μl of RIPA buffer and centrifuged at 14,000 rpm for 30 min at 4 °C. Cell lysate pre-clearing was performed by adding 40 μl of protein G-Sepharose (Amersham) to each sample for 1 h at 4 °C. The samples were then centrifuged for 3 min at 300 rpm to remove the protein G-Sepharose.

For protein immunoprecipitation (i.p.), 10 μl of the appropriate antibody was added to the cellular extract, and samples were incubated overnight at 4 °C in a constant rotation movement. The day after, 40 μl of protein G-Sepharose (Amersham) was added and kept for 1 h at 4 °C in a constant rotation movement. Precipitated proteins were collected by centrifugation, washed three times in lysis buffer, and analyzed by western blot analysis.

### Indirect immunofluorescence assay (IFA)

PaCa44 cells were grown on slides for 24 h, treated with VPA (10 mM) for 48 h, permeabilized as already described [14] and then primary polyclonal antibody against PD-L1 (1:200) (17952-1-AP) was added for 1 h at room temperature (RT). Slides were then washed with 1X PBS and incubated with the secondary antibody Fluorescein (FITC) AffiniPure Goat Anti-Rabbit IgG (H + L) (1:200) (111-095-003) for 30 min at room temperature. After 3 washes, slides were incubated with DAPI (1:5000) (Sigma Aldrich) for 1 min at RT and mounted on a slides with glycerol:PBS (1:1). Slides were analyzed using a fluorescence microscope (Olympus BX53) at 40X magnification [14].

### Antibodies

For protein immunoprecipitation (i.p.), the following antibodies were used: mouse monoclonal anti-Ac-lysine (AKL5C1) (sc-32268, Santa Cruz Biotechnology) and rabbit polyclonal anti-PD-L1 (E1L3N) (13684, Cell Signaling).

The following antibodies were used to evaluate protein expression: rabbit polyclonal anti-PD-L1 (E1L3N) (1:500) (13684, Cell Signaling Technology), mouse monoclonal anti-TRAPPC4 (C-7) (1:100) (sc-390551, Santa Cruz Biotechnology) and rabbit polyclonal anti-acetyl-Histone H3 (Lys27) (1:500) (4353, Cell Signaling). (Mouse monoclonal anti-β-actin (1:10,000) (Sigma Aldrich) and rabbit polyclonal anti-Lamin A/C (1:1000) (20 32, Cell Signaling Technology) were used as loading controls. Goat anti-mouse IgG-HRP (1:20,000) (Bethyl Laboratories, A90-116P) and goat anti-rabbit IgG-HRP (1:20,000) (Bethyl Laboratories, A120-101P, Montgomery, TX, USA) were used as secondary antibodies. All primary and secondary antibodies were diluted in a PBS + 0.1% Tween20 solution containing 3% BSA (SERVA, Reno, NV, USA).

### Chemiluminescent immunometric assay

Supernatants derived from VPA-treated and control PaCa44 cells were used in a magnetic Luminex assay with a human pre-mixed multi-analyte kit (R&D Systems Bio-Techne, Minneapolis, MN, USA), according to the manufacturer's instructions.

### Densitometric analysis

Protein bands were quantified with densitometric analysis through the ImageJ software (1.47 version, NIH, Bethesda, MD, USA).

### Statistical analysis

The results are represented as the mean ± standard deviation (SD) of at least three independent experiments. Statistical analyses were performed using GraphPad Prism^®^ software (GraphPad Software Inc., La Jolla, CA, USA) using a two-tailed Student’s t-test, one-way ANOVA and two-way ANOVA analyses to determine statistical significance. Differences were considered statistically significant when p values were * < 0.05, ** < 0.01, *** < 0.001, and **** < 0.0001).

## Results

### VPA upregulation of PD-L1 is partially counteracted by JQ-1

**Fig. 1:**
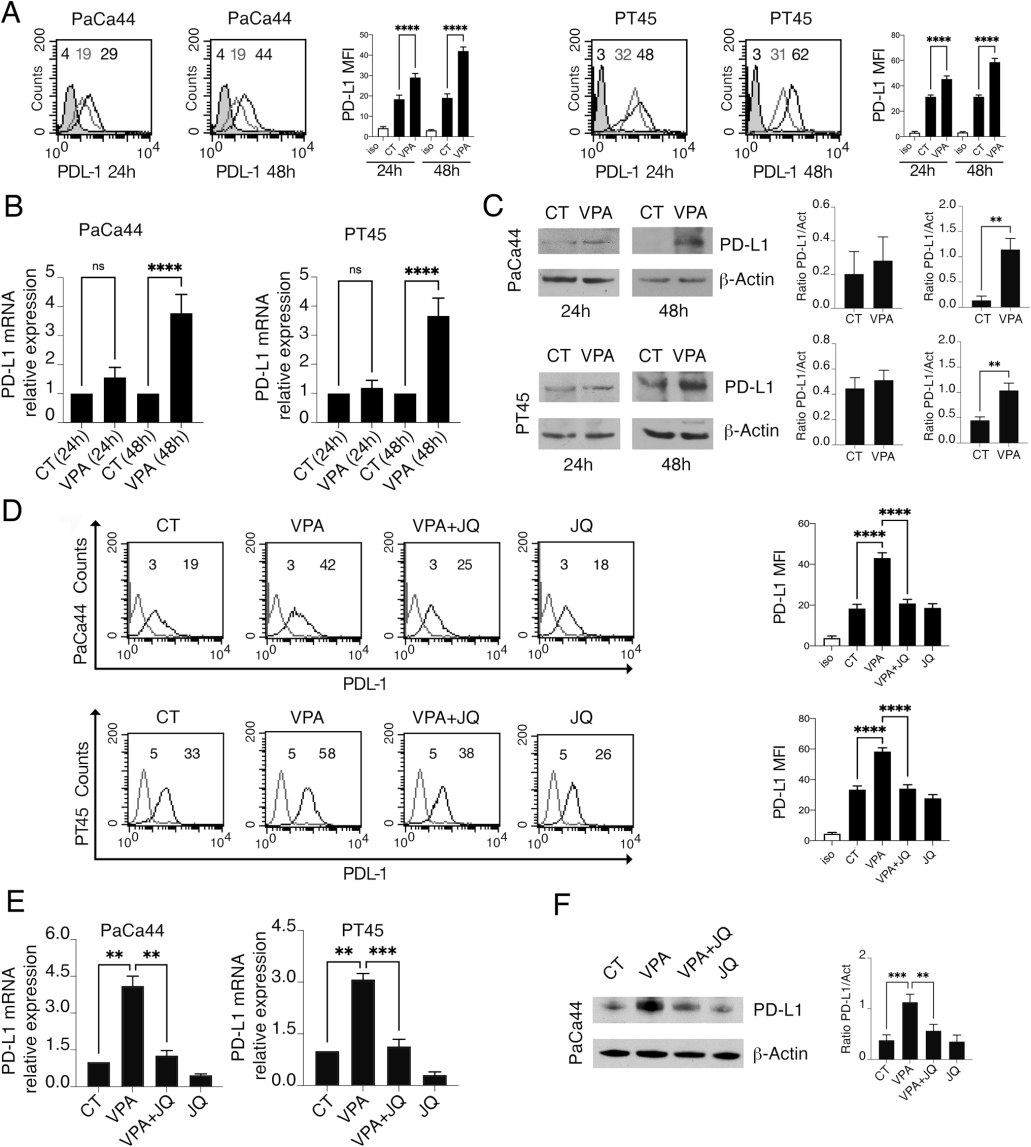
The up-regulation of PD-L1 induced by VPA is partially counteract by JQ-1. The pancreatic cell lines (PaCa44 and PT45) were treated with VPA (10 mM) for 24 and 48 h. **A** PD-L1 expression on the cell membrane was evaluated by FACS analysis (the grey line represents the untreated CT cells and the black line represents the VPA-treated cells). A representative experiment out of three and bar histograms representing the mean of the mean values of fluorescence (MFI) plus SD of three independent experiments are shown. ****p value < 0.0001. **B** mRNA expression of PD-L1 evaluated by qRT-PCR is shown. The data are expressed relative to the reference gene (β2M). Histograms represent the mRNA expression levels of PD-L1. Data are represented as the mean relative to the control plus SD. ***p < 0.001. **C** PD-L1 total expression protein was evaluated by western blot analyses. β-Actin served as the loading control. A representative experiment of three experiments is presented. The histograms representing the mean ± SD of the densitometric analysis of the ratio of PD-L1/Act are also shown. **p value < 0.01. **D** PD-L1 expression on the cell membranes of pancreatic cancer cell lines (PaCa44 and PT45) treated for 48 h with VPA (10 mM) and JQ-1 (JQ) (500 nM) singly or in combination, as evaluated by FACS analysis. A representative experiment of three experiments is presented. Bar histograms represent the mean values of fluorescence (MFI) plus the SD of at least three independent experiments. ****p value < 0.0001. **E** mRNA expression of PD-L1 as evaluated by qRT-PCR. The data are expressed relative to the reference gene (β2M). Histograms represent the mRNA expression levels of PD-L1. Data are represented as the mean relative to the control plus SD. p value **< 0.01, ***< 0.001. **F** PD-L1 total expression protein as evaluated by western blot analyses in PaCa44 cell lines. β-Actin served as the loading control. A representative experiment of three experiments is presented. Histograms representing the mean ± SD of the densitometric analysis of the ratio of PD-L1/Act are also reported. p value **< 0.01, ***< 0.001

Histone deacetylase (HDAC) inhibitors have been reported to increase histone acetylation of *PD-L1*, thereby increasing its expression [23]. Therefore, after assessing that VPA, a class I/IIa HDAC inhibitor, did increase acetylation of histone H3 (Supplementary Fig. S1), we investigated whether such treatment, could upregulate PD-L1 in two pancreatic cancer cell lines, PaCa44 and PT45. As shown in Fig. 1A, the surface expression of PD-L1 increased slightly after 24 h and was strongly upregulated after 48 h of treatment with VPA in both cell lines. Similar effects were induced by sodium phenylbutyrate (PBA), a pan-HDAC inhibitor (Supplementary Fig. S2A), suggesting that PD-L1 upregulation may represent a general effect of HDAC inhibition and increased acetylation. Conversely, treatment with nicotinamide (NAM), a Pan-Sirtuin inhibitor (class III HDAC), did not affect PD-L1 expression in pancreatic cancer cells (Supplementary Fig. S2B), suggesting that only classical HDACs may affect the expression of PD-L1. We then found that the upregulation of PD-L1 on the cell surface induced by VPA correlated with enhanced expression of this molecule, both at the mRNA (Fig. 1B) and protein (Fig. 1C) levels.

Histone acetylation increases the binding substrate for bromodomain-containing proteins (BRD), such as BRD4, which are epigenetic readers of acetylated sites. Interestingly, BRD4 has been reported to enhance the transcription of PD-L1 in different types of cancers [24–26]. Therefore, we investigated whether JQ-1, a BRD4 inhibitor, counteracted the upregulation of PD-L1 induced by VPA. As shown in Fig. 1D, PD-L1 surface expression increased by VPA was reduced by JQ-1 treatment, which also counteracted PD-L1 mRNA and protein upregulation (Fig. 1E, 1F and Supplementary Fig. S3). However, the single treatment with JQ-1 slightly influenced mRNA expression of PD-L1 and did not affect it at protein level, differently from the above reported cancers, suggesting that the effect of JQ-1 could be tumor-specific. Moreover, the treatment with JQ-1 was useful as, besides preventing PD-L1 upregulation, potentiated the cytotoxic effect of VPA (Supplementary Fig. S4). Taken together, these results suggest that increased acetylation of the PD-L1 gene by classical zinc-dependent HDACi and BRD4, which reads acetylated sites, contributes to the upregulation of PD-L1 on the cancer cell surface.

### PD-L1 expression increases in the cytoplasm of cancer cells while is reduced in the nuclear fraction following VPA treatment

**Fig. 2:**
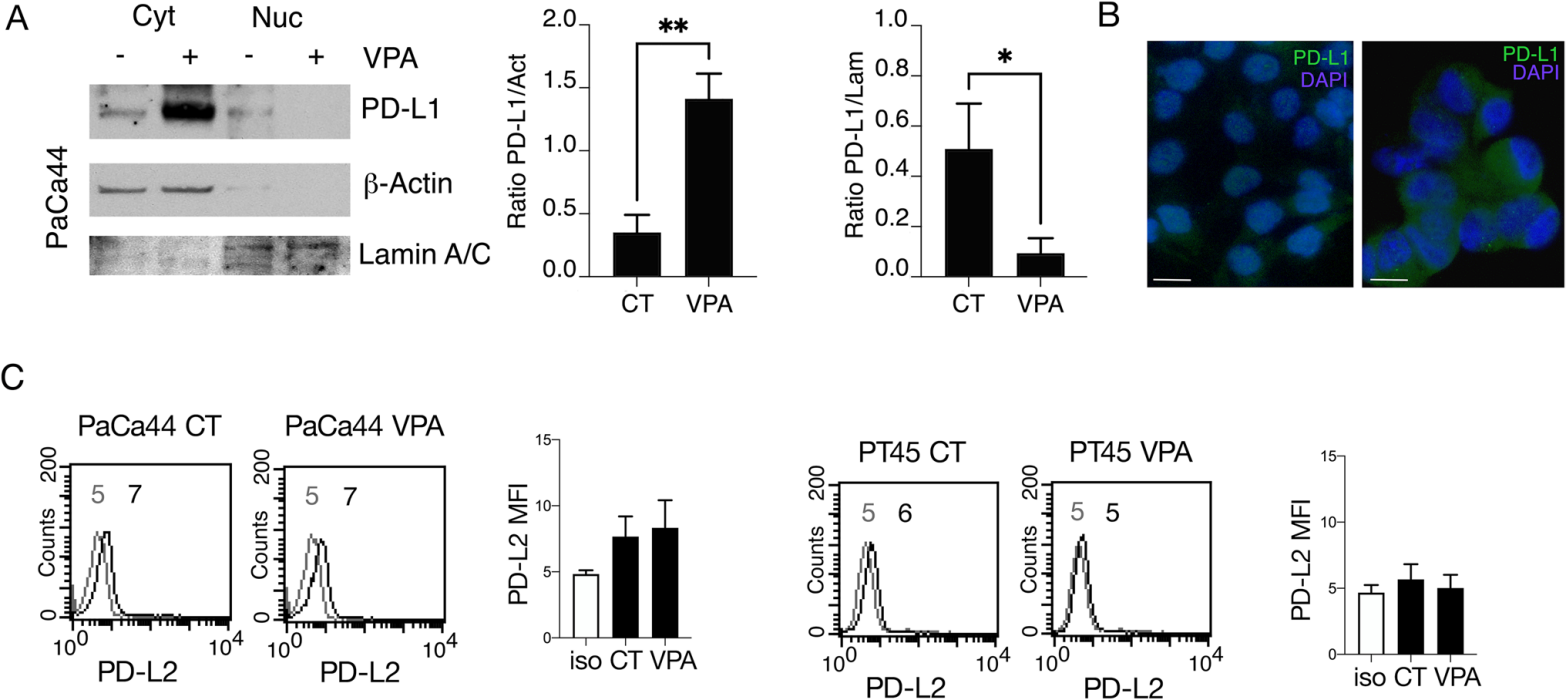
VPA-treatment upregulates PD-L1 in the cytoplasm but not in the nuclei of cancer cells. PaCa44 cells were treated with VPA (10 mM) for 48 h. **A**PD-L1 expression was evaluated in the nuclear and cytoplasmic fractions using western blot analysis. β-Actin and Lamin A/C were used as loading controls for the cytoplasmic and nuclear fractions, respectively. Histograms represent the mean ± SD of the densitometric analysis of the ratio of PD-L1/Act for the cytoplasmic fraction and the ratio of PD-L1/Lam for the nuclear fraction. p value *< 0.05, **< 0.01. **B** PD-L1 (green) localization was evaluated by IFA. DAPI (blue) was used for nuclear staining. Images were captured using fluorescence microscope at × 40 magnification. Bars = 10 μm. **C** Pancreatic cell lines (PaCa44 and PT45) treated with VPA (10 mM) for 48 h (**B**) were assessed for PD-L2 expression by FACS analysis. The bar histograms represent the mean values of fluorescence (MFI) plus the SD of three independent experiments

Evaluating the subcellular localization of PD-L1 is particularly important, as its expression on the cell surface may be blocked by anti-PD-L1 antibodies to improve the outcome of anti-cancer treatments, whereas its nuclear localization may promote transcription [1] and increase the expression of molecules that further induce immune suppression, such as PD-L2 [5]. In this study, we found that PD-L1 expression was upregulated in the cytoplasmic fraction of pancreatic cancer cells, whereas its expression in the nuclei was rather reduced following VPA treatment as evidenced by western blot analysis and by IFA (Fig. 2A, B). Accordingly, the expression of PD-L2, considered a target of PD-L1, was not affected by VPA (Fig. 2C), suggesting that VPA treatment is likely to benefit from PD-L1/PD-1 blockade therapy.

### VPA increases PD-L1 protein acetylation, enhances its interaction with TRAPPC4 and the localization on cancer cell surface

**Fig. 3:**
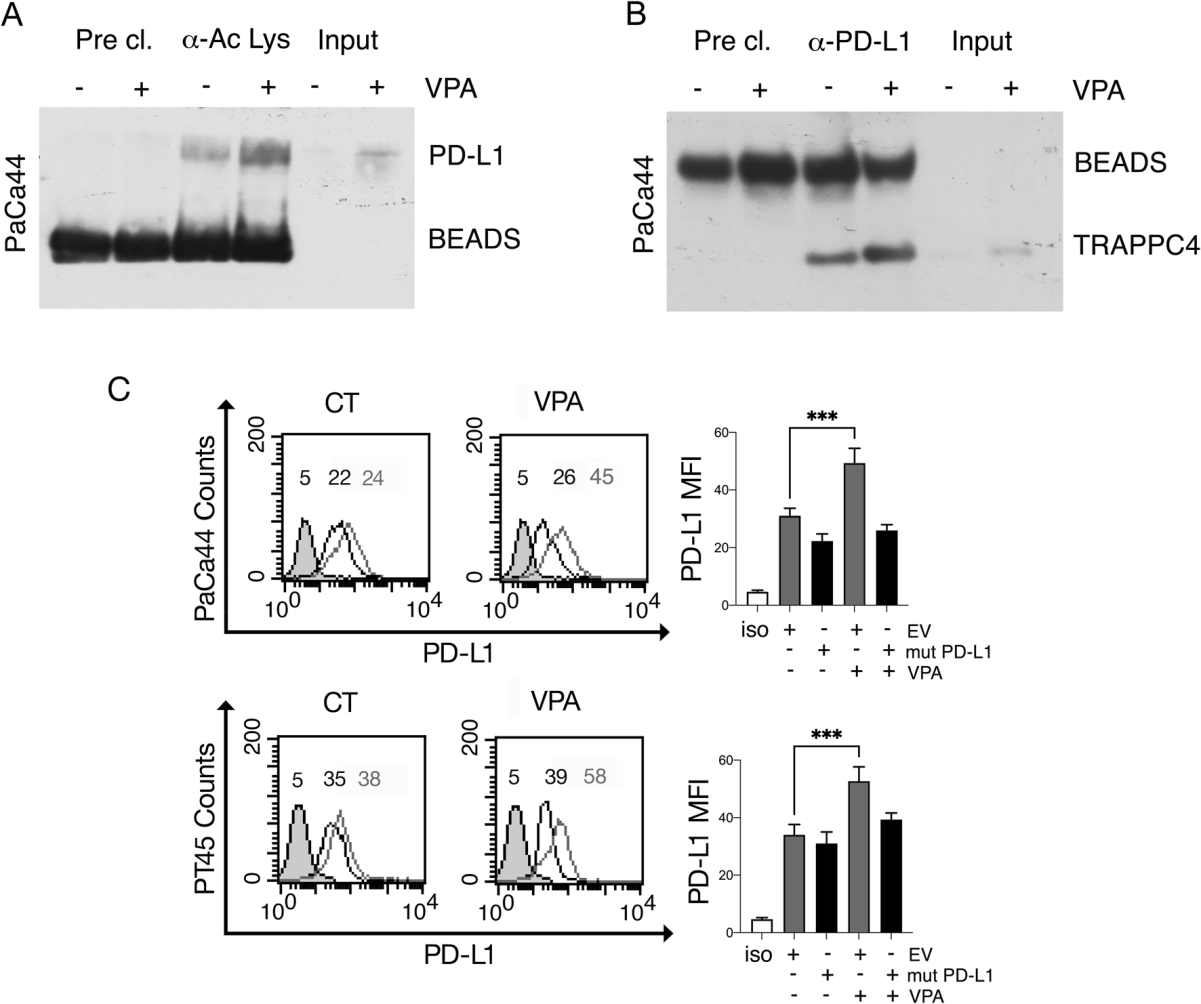
VPA treatment increases PD-L1 protein acetylation enhancing its interaction with TRAPPC4. PaCa44 cells were treated with VPA (10 mM) for 24 and 48 h. **A** PD-L1 acetylation as evaluated by western blot analysis after immunoprecipitation with anti-acetylated-lysine antibody (α-Ac Lys). **B** TRAPPC4 expression was evaluated by western blot analysis after immunoprecipitation with anti-PD-L1 antibody (α-PD-L1). As negative control we used IP w/o antibodies (Pre-cl.) and crude lysate (Input). PaCa44 and PT45 cells were transfected with PD-L1K263R (mut PD-L1) and then treated with VPA (10 mM). **C** PD-L1 expression in the cell membrane was evaluated by FACS analysis. Empty vector (EV)- transfected cells were used as the controls. The grey line represents EV-transfected cells, and the black line represents PD-L1 K263R-transfected cells. A representative experiment of three experiments is presented. Bar histograms represent the mean values of fluorescence (MFI) plus the SD of at least three independent experiments. p value ***< 0.001

It has been reported that PD-L1 protein may be acetylated at Lys 263 by p300 and deacetylated by HDAC2 and that such epigenetic modification may negatively influence its nuclear import [7]. Given that VPA inhibits HDAC2 [12], here we investigated whether this HDACi could increase PD-L1 acetylation. As shown in Fig. 3A, i.p. analysis revealed that VPA enhanced the acetylation of PD-L1 in PaCa44 cells. A recent study showed that PD-L1 interacts with TRAPPC4, a molecule involved in PD-L1 endosomal recycling to the cell membrane [4]. Here, we investigated whether the increased acetylation of PD-L1 induced by VPA could influence its binding to TRAPPC4, thereby promoting its localization on the cell surface. We found that PD-L1 bound more efficiently to TRAPPC4 following VPA-treatment (Fig. 3B), an effect that may contribute to the localization of PD-L1 on the cancer cell surface. To evaluate the role of PD-L1 acetylation in facilitating its surface expression, we transfected cancer cells with the acetylation-deficient PD-L1K263R mutant, according to a previous study [7]. As shown in Fig. 3C, PD-L1K263R transfected cells displayed reduced expression of PD-L1 on the cell surface following VPA treatment, supporting the hypothesis that acetylation of PD-L1 protein was promoting its localization on the cell surface. As control, we assessed that VPA did not increase PD-L1 protein acetylation in PD-L1K263R transfected cells, effect observed in empty vector (EV) transfected cells (Supplementary Fig. S5).

**Fig. 4:**
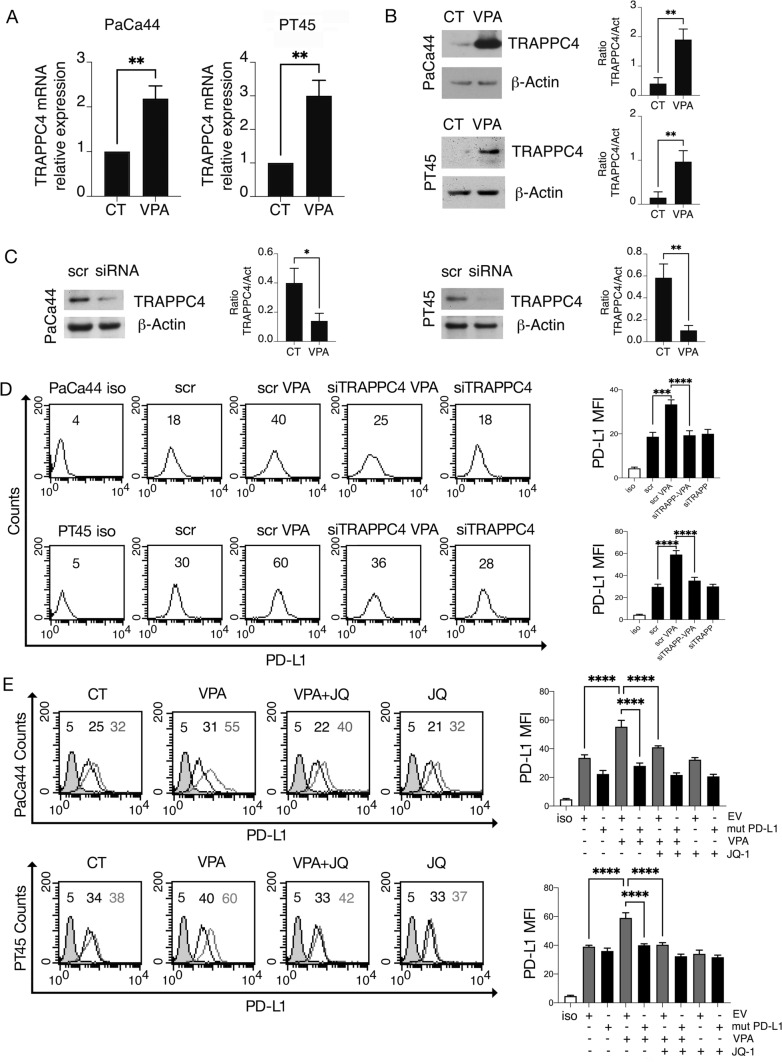
VPA upregulates TRAPPC4 expression that influences PD-L1surface localization. Pancreatic cancer cells (PaCa44 and PT45) treated with VPA (10 mM) for 48 h and analyzed for **A** mRNA expression of TRAPPC4 by qRT-PCR. The data are expressed relative to the reference gene (β2M). Histograms represent mRNA expression levels of TRAPPC4. Data are represented as the mean relative to the control plus SD. p value **< 0.01. **B** TRAPPC4 protein expression as evaluated by western blot analysis. β-Actin was used as the loading control. Histograms represent the mean ± SD of the densitometric analysis of the TRAPPC4/Act ratio. p value **< 0.01. PaCa44 and PT45 cells were knocked down for TRAPPC4 with specific siRNA (siTRAPPC4) and then treated with VPA (10 mM) were analyzed for **C** TRAPPC4 expression by western blot analysis. β-Actin was used as the loading control. Control cells were transfected with the control siRNA (scr). Histograms represent the mean ± SD of the densitometric analysis of the TRAPPC4/Act ratio. p value *< 0.05, **< 0.01. **D** PD-L1 expression on the cell membrane, evaluated by FACS analysis. A representative experiment out of three and bar histograms representing the mean values of fluorescence (MFI) plus the SD of at least three independent experiments are shown. p value ***< 0.001, ****< 0.0001. **E** Pancreatic cancer cells (PaCa44 and PT45) transfected with PD-L1 K263R plasmid and treated with VPA (10 mM) and/or JQ-1 (500 nM) for 48 h were analyzed by FACS for PD-L1 expression on the cell membrane. Empty vector-transfected cells were used as a control (EV). The grey line represents EV-transfected cells, and the black line represents PD-L1K263R-transfected cells. A representative experiment of three experiments is presented. The bar histograms represent the mean values of fluorescence (MFI) plus the SD of three independent experiments. p value ****< 0.0001

Notably, VPA enhanced the expression of TRAPPC4 at both the mRNA and protein levels (Fig. 4A, B), which increased the amount of protein available for binding to PD-L1. The involvement of TRAPPC4 in promoting the surface localization of PD-L1 in VPA-treated cells was assessed by its silencing (Fig. 4C), which partially prevented surface upregulation of PD-L1 (Fig. 4D). Finally, when the combination treatment with VPA/JQ-1 was used against pancreatic cancer cells transfected with acetylation-deficient PD-L1K263R, it completely prevented the upregulation of PD-L1 induced by VPA on the cell surface (Fig. 4E). These results suggest that acetylation of PD-L1 enhances the binding toTRAPPC4 and this effect, together with PD-L1 upregulation, may contribute to its increased expression on cancer cells surface following VPA treatment.

### Cytokines released by VPA-treated cancer cells do not affect PD-L1 expression on macrophage surface

**Fig. 5:**
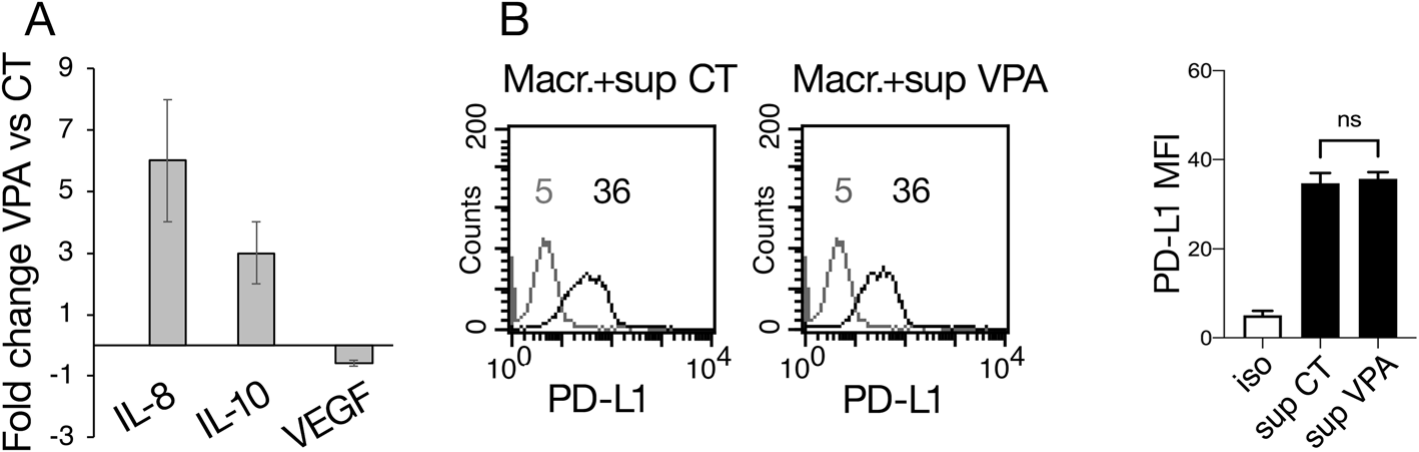
VPA alters the release of cytokines without affecting PD-L1 expression on macrophage surface. **A** Supernatants collected by PaCa44 cell treatment with VPA (10 mM) after 48 h of treatment were analyzed for cytokines release as evaluated by Magnetic Luminex Assay. Histograms representing the mean plus SD out of at least three different experiments. **B** Macrophages, derived from PBMCs purification, cultured with 25% vol/vol of supernatant of VPA-treated or untreated cells for 24 h were analyzed for PD-L1 cell membrane expression by FACS analysis. A representative experiment out of three is shown. Bar histograms represent the means of the mean values of fluorescence (MFI) plus SD of at least three independent experiments

Some cytokines, such as IL-8 and IL-10, may counteract the efficacy of PD-L1/PD-1 blockade therapy [27, 28] and affect the expression of PD-L1 on the surface of tumor-associated macrophages (TAM) [3], the most abundant immune cells in the tumor microenvironment (TME). In this study, we evaluated the release of cytokines in the supernatant of VPA-treated pancreatic cancer cells and found that VPA increased the release of IL-8 and to a lesser extent than IL-10, while the production of VEGF was reduced compared to that in untreated cancer cells (Fig. 5A). These cytokines have been reported to negatively influence PD-L1/PD-1 blockade therapy [29] and therefore this effect should be considered before approaching the treatment PD-L1/PD-1 blockade therapy in combination with VPA. Finally, we investigated whether the supernatant of VPA-treated pancreatic cancer cells could affect PD-L1 expression on the surface of macrophages. As shown in Fig. 5B, the VPA-supernatant slightly influenced the expression of PD-L1 on the surface of macrophages, in comparison to those exposed to the supernatant of untreated pancreatic cancer cells, which suggests that such an immunosuppressive effect on macrophages was not induced by treating cancer cells with VPA.

## Discussion

Epigenetic modifications, such as acetylation and the activity of bromodomain and extra-terminal (BET) proteins, which read acetylated lysine on histone and other nuclear proteins, may influence the expression and nuclear localization of PD-L1 [1, 7, 24, 30]. In this study, we show that VPA upregulated PD-L1 on the surface of pancreatic cancer cells, similarly to what reported for thyroid tumor cells [31]. To this effect contributed the increased transcription, which was counteracted by the BRD4 inhibitor JQ-1, and the increased acetylation of PD-L1 protein. In line with this effect, a transcriptional inhibition of PD-L1 expression by BET inhibitors has been previously reported [24]. In this study, we also show that the increased acetylation of PD-L1 induced by VPA increased the efficiency of its binding to TRAPPC4, a membrane-trafficking molecule promoting PD-L1 endosomal recycling to the cell membrane [4]. This finding suggests that acetylation may contribute to the localization of PD-L1 on cancer cell surface, in addition to reducing its nuclear import, as reported in a previous study [7]. As in this above-mentioned study, here we assessed the role of PD-L1 protein acetylation by transfecting pancreatic cancer cells with the acetylation-deficient PD-L1K263R mutant, which led to a reduced surface expression of PD-L1 in cancer cells treated with VPA. However, complete prevention of PD-L1 surface upregulation in VPA-treated cells was achieved by treating pancreatic cancer cells transfected with acetylation-deficient PD-L1K263 and VPA in combination with JQ-1 combination. This further indicates that both transcriptional increase and PD-L1 protein acetylation played a role in the increase of its surface expression. Lysine acetylation has been reported to influence the stability of several molecules involved in physiological and pathological processes [32], such as anti-apoptotic myeloid cell leukemia 1 (MCL1) [33] and transcription factor EB (TFEB) [30]. Therefore, VPA could increase the expression level of PD-L1 also by enhancing its stability. Regarding TRAPPC4, it is important to highlight that its expression was upregulated by VPA, which, in addition to increasing the amount of protein able to bind PD-L1 and promote its surface localization, could represent a mechanism of resistance to VPA-induced cytotoxicity. Indeed, it has been reported that TRAPPC4 can promote colorectal carcinogenesis by activating Wnt signaling [34] or sustaining gastric cancer survival through extracellular signal-regulated protein kinase (ERK) signaling [35]. Notably, Wnt [36] and ERK1/2 [37] are among the pathways that may regulate PD-L1 expression. Exploring the impact of anti-cancer treatments on PD-L1 expression and on the regulation of its subcellular localization is particularly important as, when over-expressed on plasma-membrane, it can be targeted by therapeutic antibodies such as atezolizumab, avelumab or durvalumab, to improve the outcome of anti-cancer therapies. Such humanized antibodies help prevent the interaction of PD-L1 with PD-1, expressed by T cells, and hopefully restore the T cell-based anti-cancer immune response, which is essential to fight cancer [38]. Although high expression of PD-L1 on the cell surface is considered a biomarker that predicts the success of anti-PD-L1/PD-1 blockade therapies, there are mechanisms that have not yet been completely elucidated, which may limit its success in clinical practice and are worth further investigation [39]. One of these factors is the localization of PD-L1 in the nuclear compartment, where it acts as a transcription factor and may enhance the expression of other immunosuppressive molecules, such as PD-L2 and B7H3 [1]. Interestingly, in this study, we found that VPA did not lead to PD-L2 upregulation, even if HDACi have been reported to do so in melanoma’s cells [23], suggesting that this effect may varies depending on the tumor types. However, VPA increased the production of IL-8 and, to a lesser extent, IL-10, which represents another mechanism that may decrease the efficacy of the PD-L1/PD-1 blockade strategy [40]. To overcome this problem, clinical trials are ongoing to combine anti-IL8 or anti-IL10 antibodies with anti-PD-L1/PD-1 blockade [28, 29, 40]. In contrast to IL-8 and IL-10, VPA slightly reduced the production of VEGF, a cytokine that displays pro-angiogenetic and immune suppressive effects, and is also able to reduce the benefit of anti-PD-L1 therapies [41].

Finally, we found that, despite dysregulating the release of cytokines that may affect PD-L1 expression [3], the supernatant of VPA-treated pancreatic cancer cells did not alter PD-L1 expression on the surface of macrophages. This is important, as macrophages play an essential role in carcinogenesis, being able to either fight or support tumor growth, mainly depending on their M1 or M2 polarization [42]. We previously showed that VPA did not influence the function of another immune cell type playing a key role in anti-cancer immune response, dendritic cells, which further encourages the use of VPA as a safe molecule [13]. Interestingly, VPA has also been shown not to alter PD-L1 surface expression on Myeloid-derived suppressor cells (MDSCs) [43]. However, despite these observations that suggest that VPA may be quite safe towards the cells of the immune system, strategies aimed at encapsulating it in liposomes or nanoparticles, which could be covered with specific antibodies able to target antigens expressed on tumor cells, are in progress in our laboratory.

The immune function of dendritic cells and macrophages may be influenced, among other factors, by the cytokines present in the tumor milieu [44] as well as by their surface expression of PD-L1 [45].

**Fig. 6:**
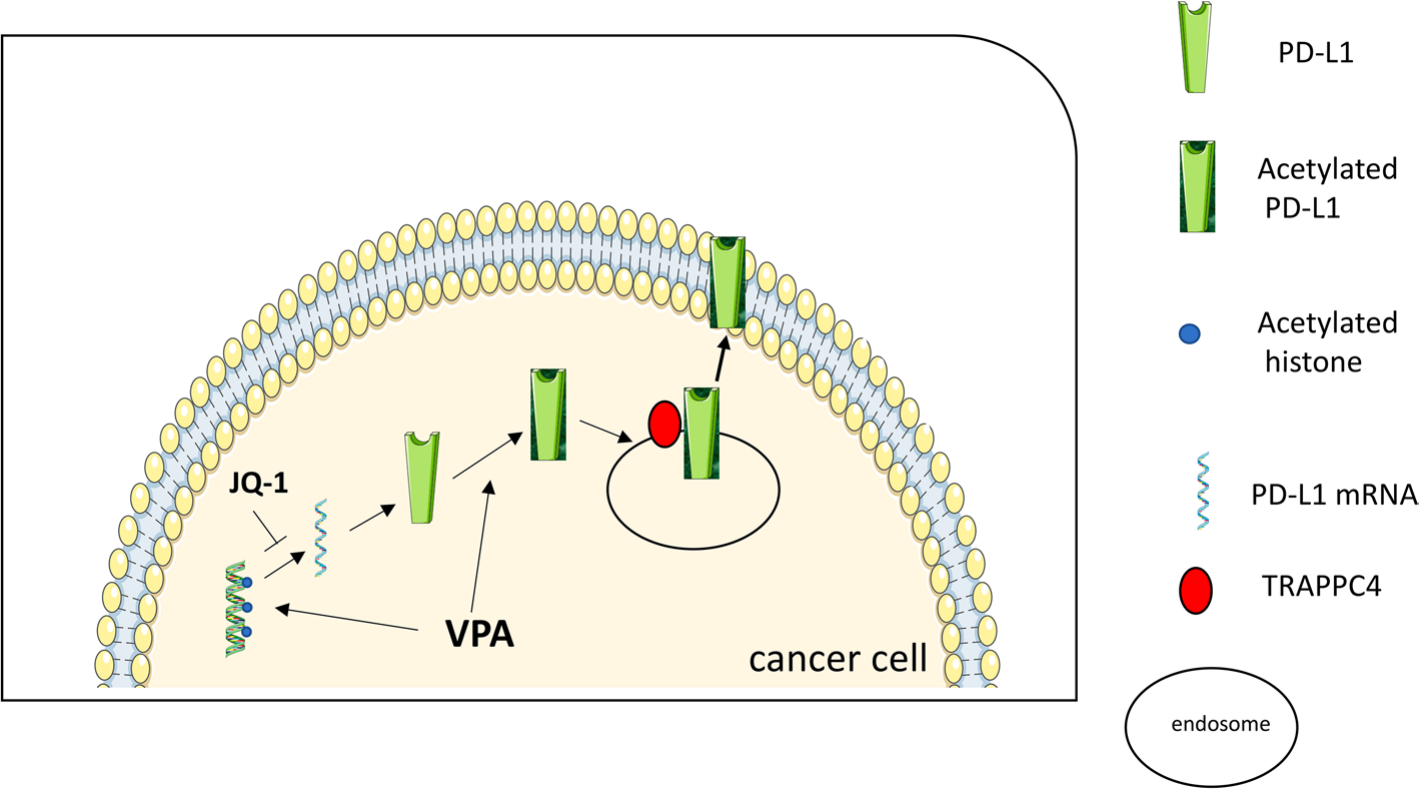
Schematic representation of effects induced by VPA on PD-L1 expression and localization in pancreatic cancer cells

In conclusion, this study shows that acetylation induced by VPA regulates the expression and subcellular localization of PD-L1, upregulating it on the cell surface while reducing its expression in the nuclei of pancreatic cancer cells, avoiding the transcription of other suppressive molecules such as PD-L2. This study also suggests that the effects induced by VPA in terms of PD-L1 expression could be reduced by JQ-1 treatment, which also increased VPA cytotoxicity, highlighting that the use of such a combination may be promising to treat pancreatic cancer (Fig. 6). The search for anti-cancer strategies that offer the possibility to reduce cancer cell survival and concomitantly counteract cancer-induced immune dysfunction and co-opt the immune system in fighting cancer remains a challenge, particularly for the treatment of cancers characterized by aggressive behavior and poor response to therapies, such as pancreatic cancer.

Strategies able to manipulate the acetylation of PD-L1 and block PD-L1/PD-1 interaction could be useful in combination with VPA, although this drug increased the production of cytokines such as IL-8, which should be neutralized to improve the outcome of this treatment.

### Supplementary Information

Below is the link to the electronic supplementary material.

Supplementary file 1 (TIF 2496 KB)

Supplementary Figure 1 VPA treatment increases histone-H3 acetylation in time-dependent manner. PaCa44 cells were treated with VPA (10 mM) and histone-H3 acetylation was evaluated by western blot analysis at 24 and 48 h of treatment. β-Actin was used as the loading control. Histograms represent the mean ± SD of the densitometric analysis of the histone H3/Act ratio. p value **< 0.01, ****< 0.0001 (TIF 297 KB)

Supplementary Figure 2 Impact on PD-L1 cell surface expression mediated by PBA and NAM. PaCa44 and PT45 cells treated with PBA (10 mM) for 48 h were analyzed for A) PD-L1 expression in the cell membrane by FACS analysis. A representative experiment of three experiments is presented. Bar histograms represent the mean values of fluorescence (MFI) plus the SD of at least three independent experiments. p value **< 0.01 PaCa44 and PT45 cells were treated with nicotinamide (NAM) (15 mM) for 48 h and B) analyzed for PD-L1 expression in the cell membrane by FACS analysis. A representative experiment out of three and bar histograms representing the means of the mean values of fluorescence (MFI) plus SD of three independent experiments are shown (TIF 575 KB)

Supplementary Figure 3 Representative images showing live cells gated (R1) and analyzed for PD-L1 expression in SSC vs FCS density plot (TIF 699 KB)

Supplementary Figure 4 PaCa44 and PT45 treated for 48 h with VPA (10 mM) and JQ-1 (JQ) (500 nM) singly or in combination were analyzed for cells survival. Histograms representing the mean of the percentage plus S. D. of cell viability, as evaluated by Trypan Blue assay following the indicated treatment. p value *< 0.05, ***< 0.001, ****< 0.0001 (TIF 715 KB)

Supplementary Figure 5 PD-L1 acetylation did not increased in transfected cells exposed to VPA. PaCa44 cells were transfected with PD-L1 K263R plasmid or Empty vector (EV) and treated with VPA (10 mM) B) protein acetylation was evaluated by western blot using anti-Acetylated Lysine antibody after immunoprecipitation with anti-PD-L1 antibody (α-PD-L1). As negative control we used IP w/o antibodies (Pre-cl.) and crude lysate (Input) (TIF 641 KB)

## Data Availability

Research data are stored in an institutional repository and will be shared upon reasonable request to the corresponding author.
