# Supplementary figures and images for: Acetylation increases expression, interaction with TRAPPC4 and surface localization of PD-L1
Source: Discov Oncol. 2023 Aug 21;14:152. doi: 10.1007/s12672-023-00766-4 (PMC10442048; doi:10.1007/s12672-023-00766-4)

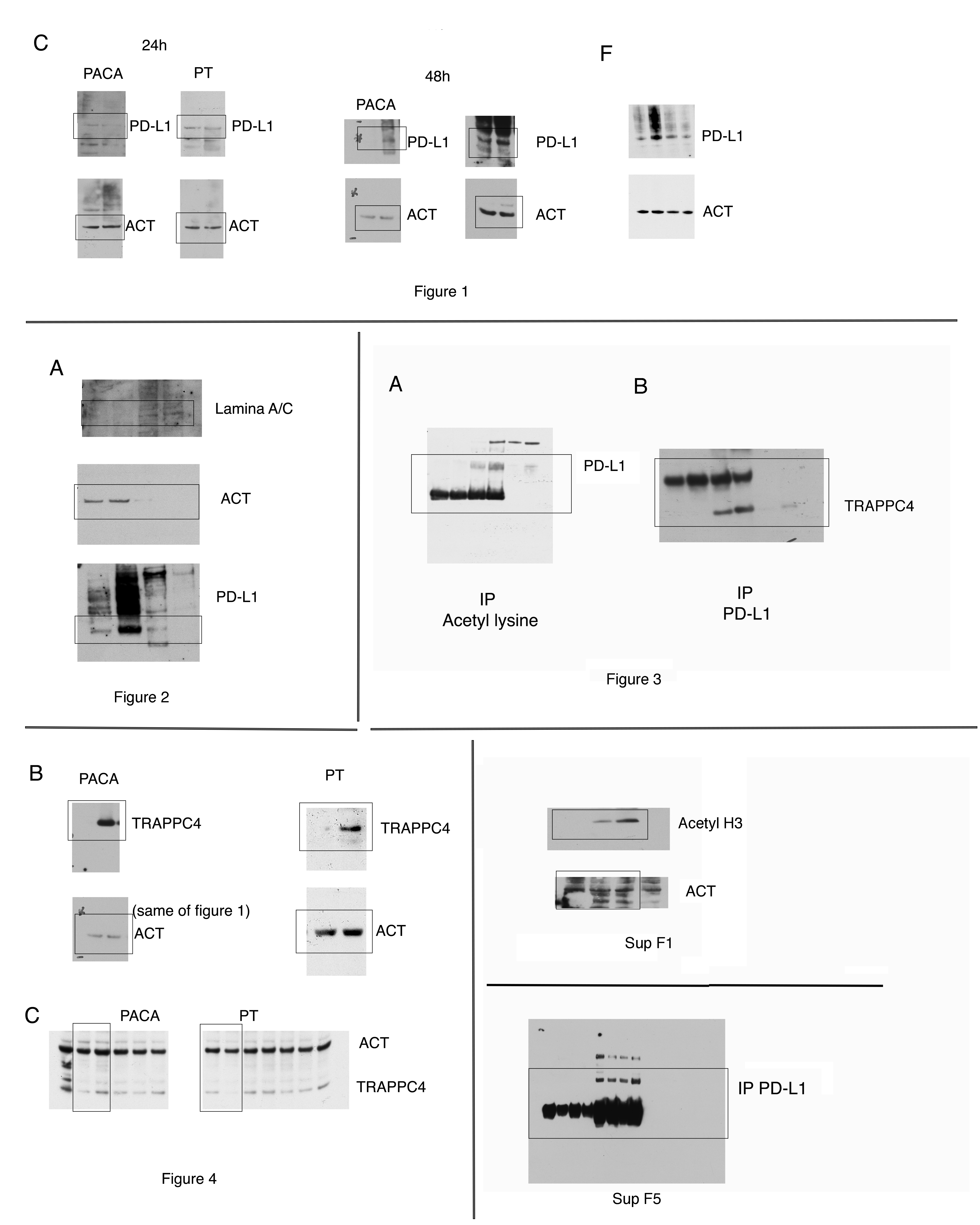

Supplement: Supplementary file 1 — (TIF 2496 KB) [file 12672_2023_766_MOESM1_ESM.tif]

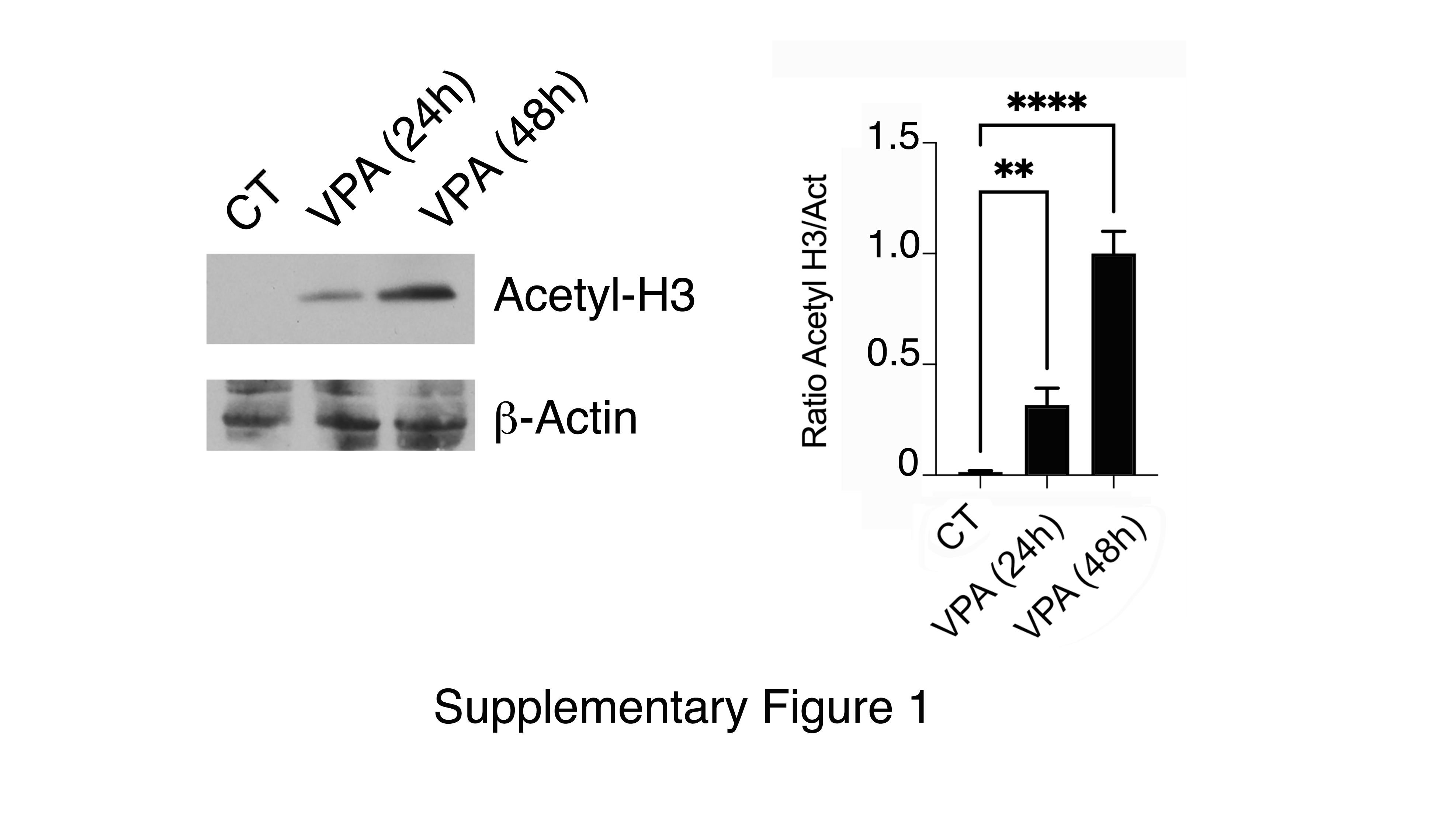

Supplement: Supplementary file 2 — VPA treatment increases histone-H3 acetylation in time-dependent manner. PaCa44 cells were treated with VPA (10 mM) and histone-H3 acetylation was evaluated by western blot analysis at 24 and 48 h of treatment. β-Actin was used as the loading control. Histograms represent the mean ± SD of the densitometric analysis of the histone H3/Act ratio. p value **< 0.01, ****< 0.0001 (TIF 297 KB) [file 12672_2023_766_MOESM2_ESM.tif]

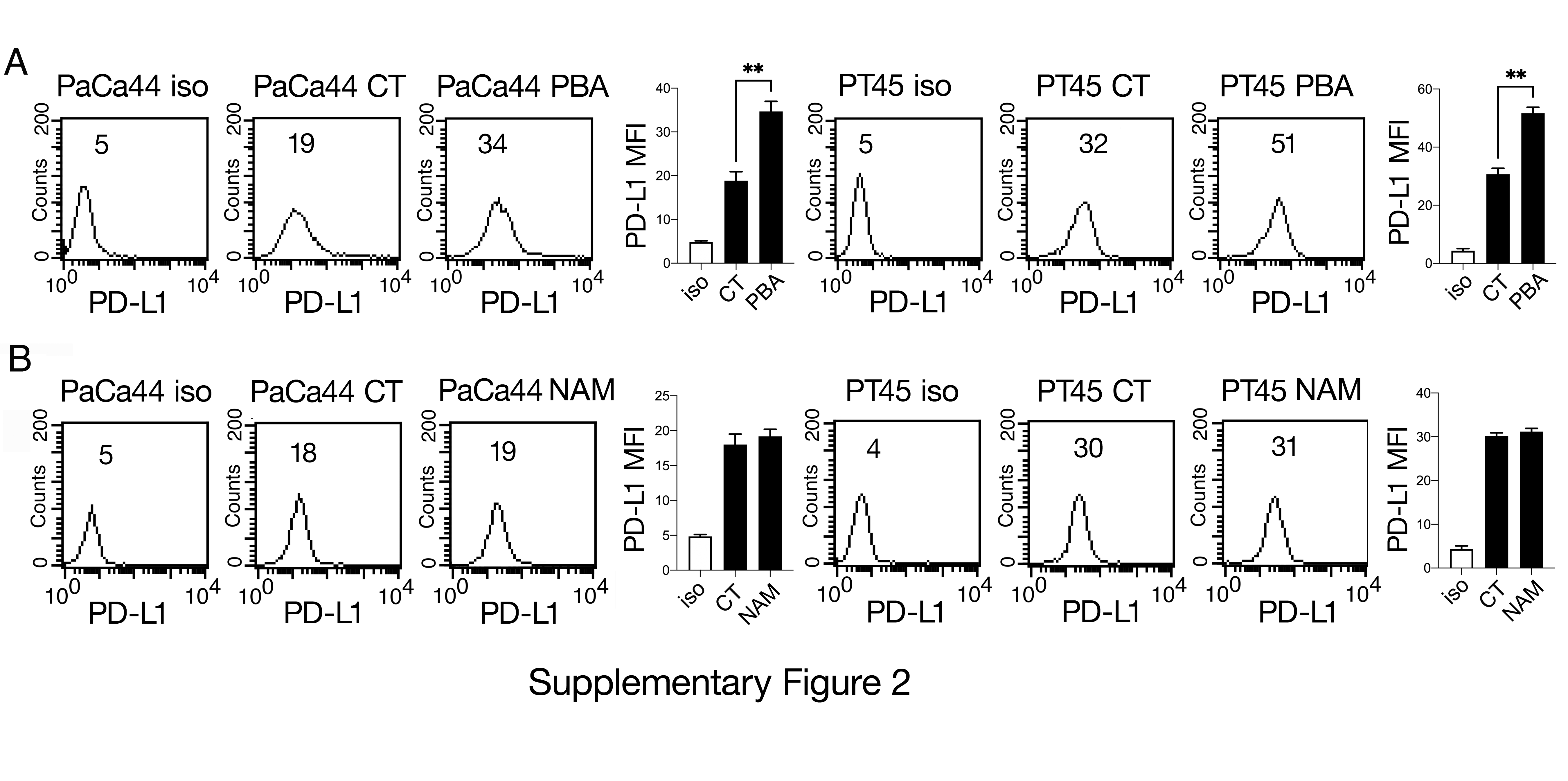

Supplement: Supplementary file 3 — Impact on PD-L1 cell surface expression mediated by PBA and NAM. PaCa44 and PT45 cells treated with PBA (10 mM) for 48 h were analyzed for A) PD-L1 expression in the cell membrane by FACS analysis. A representative experiment of three experiments is presented. Bar histograms represent the mean values of fluorescence (MFI) plus the SD of at least three independent experiments. p value **< 0.01 PaCa44 and PT45 cells were treated with nicotinamide (NAM) (15 mM) for 48 h and B) analyzed for PD-L1 expression in the cell membrane by FACS analysis. A representative experiment out of three and bar histograms representing the means of the mean values of fluorescence (MFI) plus SD of three independent experiments are shown (TIF 575 KB) [file 12672_2023_766_MOESM3_ESM.tif]

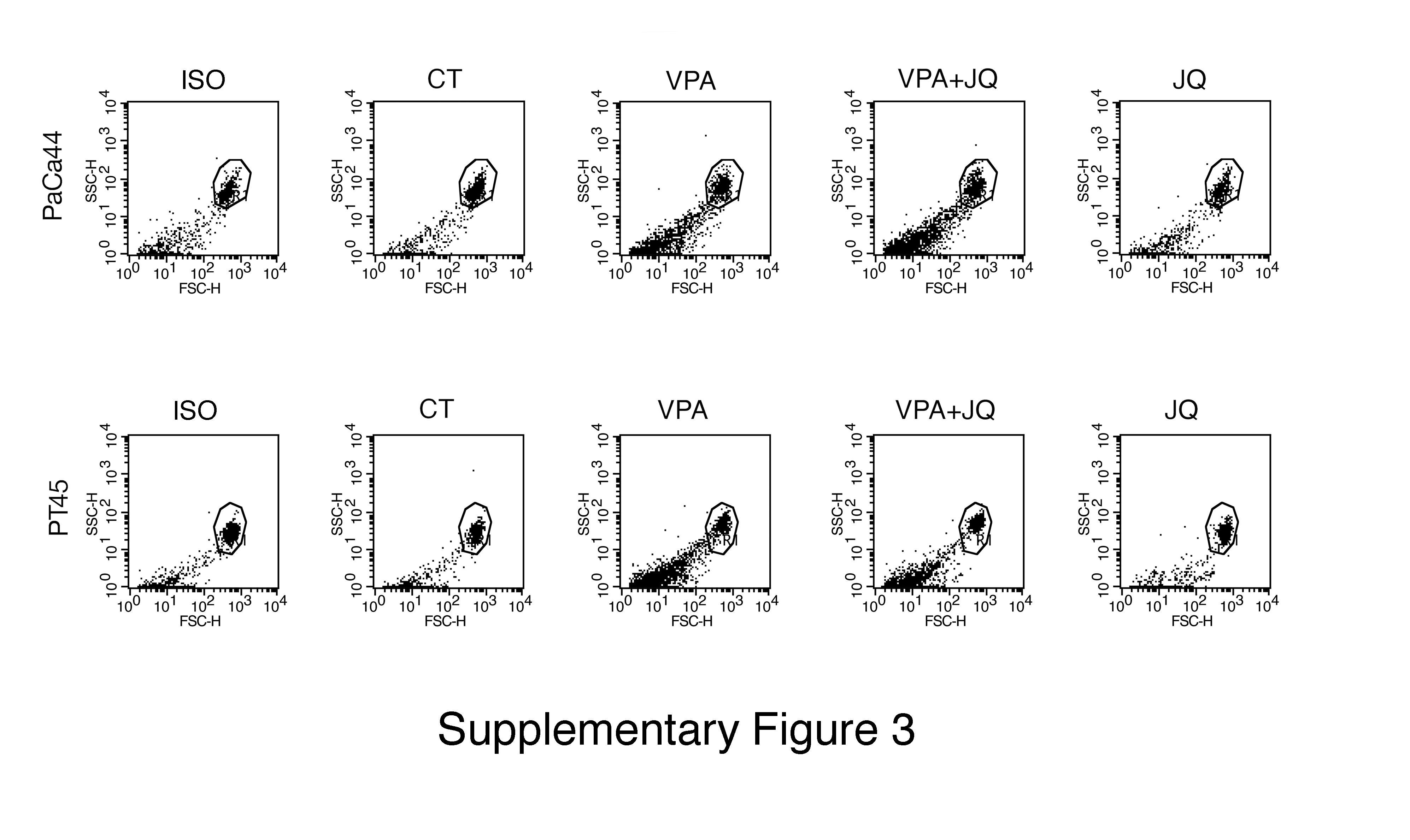

Supplement: Supplementary file 4 — Representative images showing live cells gated (R1) and analyzed for PD-L1 expression in SSC vs FCS density plot (TIF 699 KB) [file 12672_2023_766_MOESM4_ESM.tif]

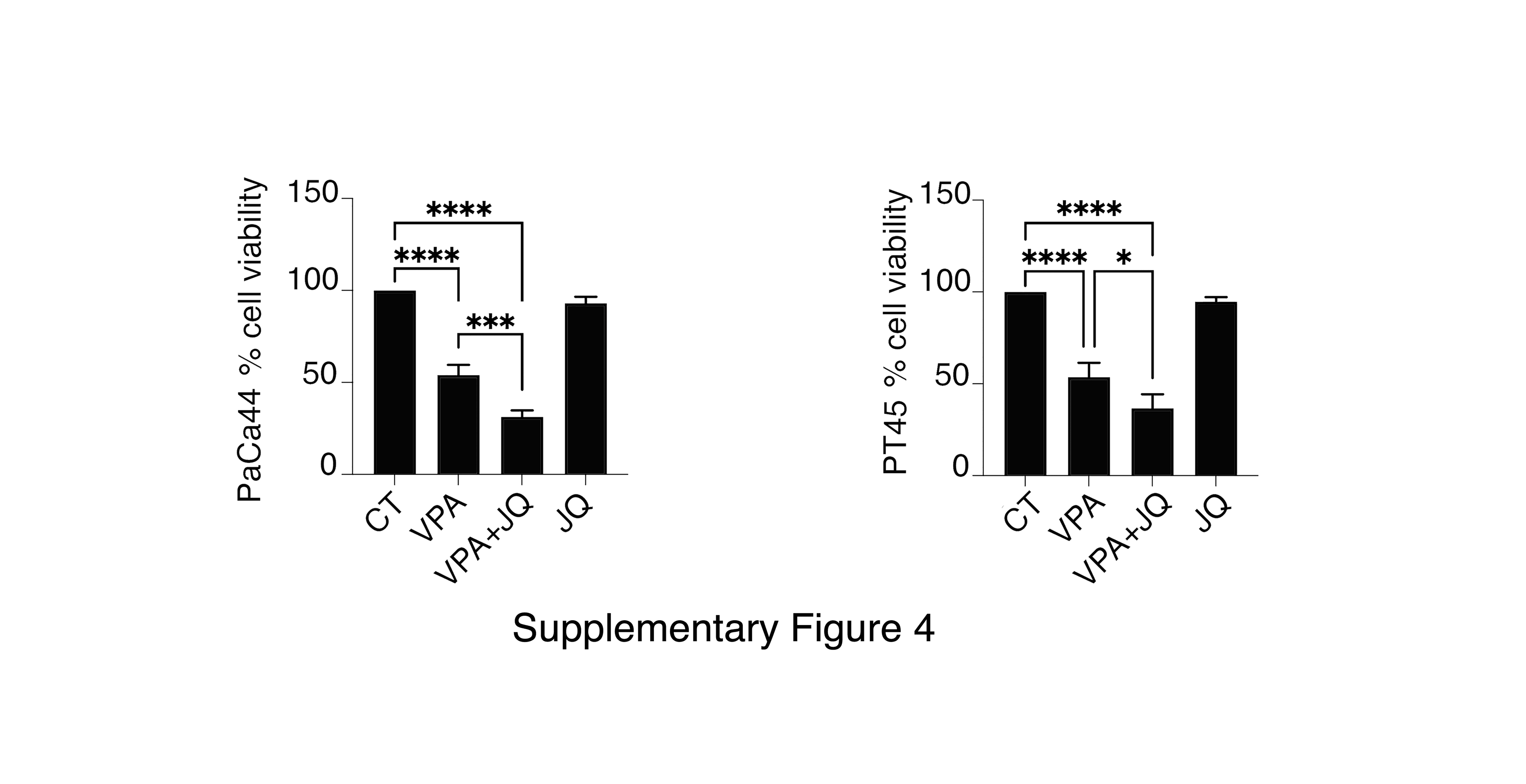

Supplement: Supplementary file 5 — PaCa44 and PT45 treated for 48 h with VPA (10 mM) and JQ-1 (JQ) (500 nM) singly or in combination were analyzed for cells survival. Histograms representing the mean of the percentage plus S. D. of cell viability, as evaluated by Trypan Blue assay following the indicated treatment. p value *< 0.05, ***< 0.001, ****< 0.0001 (TIF 715 KB) [file 12672_2023_766_MOESM5_ESM.tif]

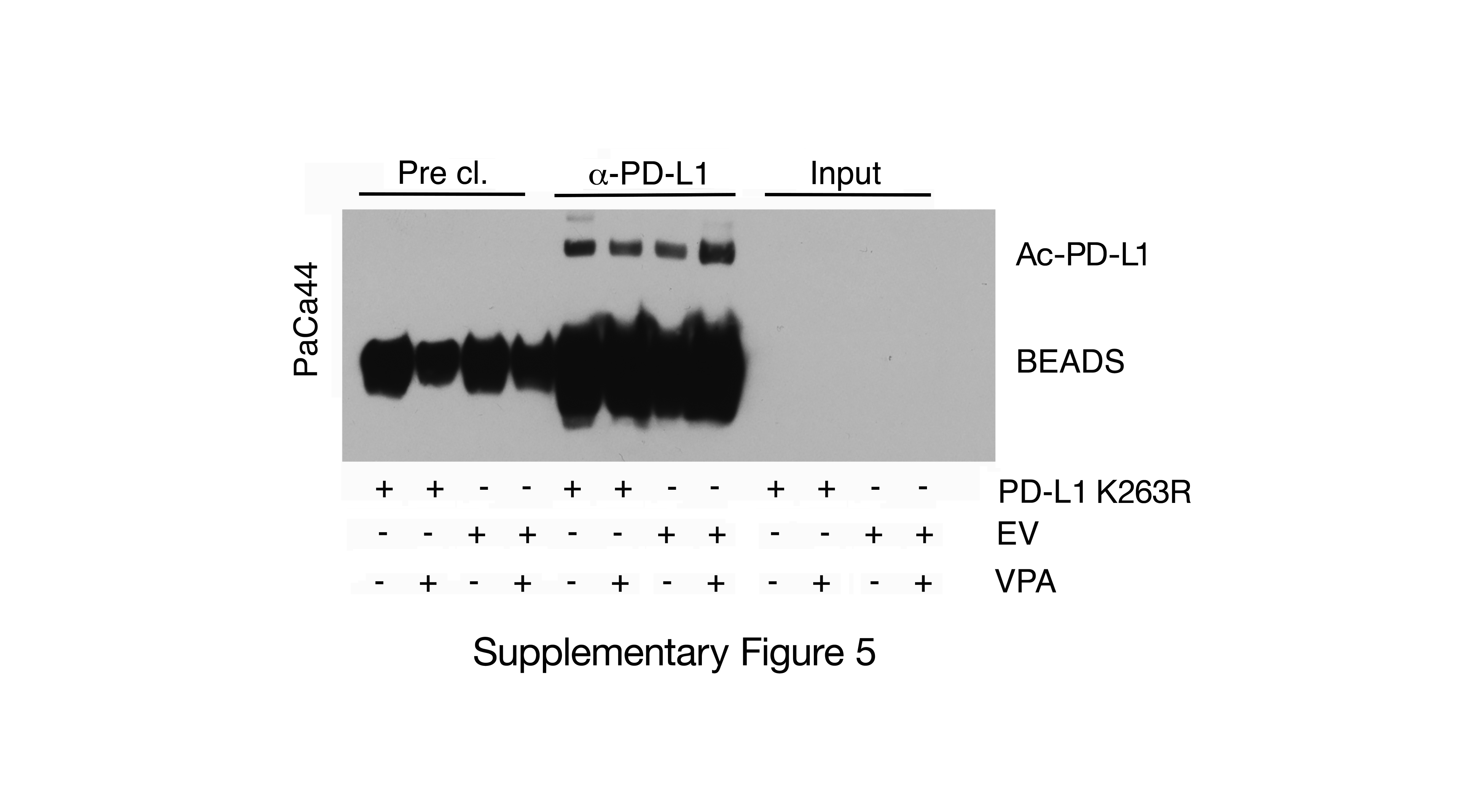

Supplement: Supplementary file 6 — PD-L1 acetylation did not increased in transfected cells exposed to VPA. PaCa44 cells were transfected with PD-L1 K263R plasmid or Empty vector (EV) and treated with VPA (10 mM) B) protein acetylation was evaluated by western blot using anti-Acetylated Lysine antibody after immunoprecipitation with anti-PD-L1 antibody (α-PD-L1). As negative control we used IP w/o antibodies (Pre-cl.) and crude lysate (Input) (TIF 641 KB) [file 12672_2023_766_MOESM6_ESM.tif]
